# Magnetic single atom catalyst in C_2_N to induce adsorption selectivity toward oxidizing gases

**DOI:** 10.1038/s41598-021-95474-w

**Published:** 2021-08-04

**Authors:** Muhammad Mushtaq, Nacir Tit

**Affiliations:** 1 Department of Physics, College of Science, UAE University, P.O. Box 15551, Al-Ain, United Arab Emirates; 2 National Water and Energy Center, UAE University, P.O. Box 15551, Al-Ain, United Arab Emirates

**Keywords:** Energy science and technology, Materials science, Nanoscience and technology, Physics

## Abstract

Density functional theory (DFT) method is used to study the effect of single-atom catalyst (SAC) of Mn embedded in C_2_N nanoribbon (C_2_N-NR) on the adsorption properties as an attempt to achieve selectivity. Many gases (e.g., CO, CO_2_, H_2_, H_2_O, H_2_S, N_2_ and O_2_) of interest to energy and environmental applications were tested. The results show that SAC-Mn alters chemisorption processes with all gas molecules except N_2_. Clear adsorption selectivity is obtained towards oxidizing CO, CO_2_ and O_2_ molecules as evidenced by the enhancements in binding energy and charge transfer and the reduction in magnetization. While the SAC-Mn contributes predominantly to Fermi-energy region with spin-down states, the strong binding to oxidizing molecules introduces there more spin-up states to compromise and reduce the magnetization. Hence, C_2_N-NR:Mn is proposed to be used as platform for gas sensor (if combined with magnetic sensor) to yield high selectivity toward these latter gases.

## Introduction

The discovery of graphene by Geim and Novoselov^1^ has opened an immense field of 2D systems with promising high-tech applications rich with excitement and discoveries and has effectively boosted the technology toward the fabrication of nano-devices^2^. Actually, soon after the Nobel prize ceremony in 2010, very fast development has been taking place and the dream of the inventors is getting realized day after day. Graphene becomes a special and a unique material known with its great flexibility and multi-functionality. For instance: **(i)** In gas-sensing: The inventors anticipated graphene to take the lead to have an extraordinary sensitivity to the limit of detecting individual gas molecules^3^. Indeed, room temperature detections of many gases, which used to be challenging tasks using metal-oxides (e.g., SnO_2_ and ZnO), became achievable using graphene and related 2D materials with high sensitivity at order of ppb and thus reaching outstanding selectivity^4,5^. **(ii)** In nano-electronics: It was considered for post-silicon nano-electronics such as graphene-based field-effect transistor (G-FET)^6,7^. **(iii)** In photonics: The functionalization of graphene induced energy gap in it and made it rather concurrent for diversity of photonic applications ranging from dye-sensitized solar cells to LED to touch screens to photodetectors and to ultrafast lasers^8–13^. **(iv)** In spintronics: Even though with the apparent lack of magnetism and the weak of spin–orbit coupling, magnetic impurities in graphene find unusual host for their spin relaxation to promote graphene to become distinct material for spin transport and spintronic devices^14,15^. **(v)** In biomedicine: Graphene finds broad field of applications ranging from drug delivery to cancer therapies to bio-sensing (i.e., to detect glucose, dopamine, protein and DNA)^16,17^. **(vi)** In strain sensors: Functionalization of graphene (e.g., graphene oxide) can reduce its stiffness to become suitable for strain sensing^18,19^.

Another more recent breakthrough was achieved in 2015 by the successful synthesis of new material based on the nitrogenated holey graphene “C_2_N” by Mahmood and co-workers using a bottom-up wet chemical reaction method^20^. C_2_N not only has a uniform distribution of holes but each hole is bordered with aromatic nitrogen atoms to widen the energy gap between the valence and conduction bands and, consequently, making itself useful for semiconducting applications (i.e., Eg ≈ 1.90 eV)^20^. This novel material has been proven to be very thermodynamically stable and having a large uptake capacity to adapt embedding metal catalysts in its holes that can trigger either high selectivity toward toxic gases at room temperature (e.g., in case of using transition metal catalyst) or enhanced uptake capacity in case of metal-ion battery (MIB) applications (e.g., in using light metal atoms like: Li, Na, K, Ca)^21–25^. Furthermore, the downsizing of these metal nanostructures to singly-dispersive metal atoms would be highly desirable for maximizing the efficiency of catalytically active metal site^26–28^. The single-atom catalyst (SAC) and double-atom catalyst (DAC) nanostructures have been experimentally achieved in metal oxides and in C_2_N and shown very enhanced catalytic activity towards several toxic gas reduction reactions (e.g., CO_x_, NO_x_ and SO_x_) of environmental concern^29–31^. The successful syntheses of SAC and DAC in C_2_N also inspired and triggered many theoretical efforts to study the corresponding reaction mechanisms^32–34^.

On the computational side, many researchers reported the importance of utilization of transition metal (TM) atoms as catalysts for gas-sensing applications. For instance, Zhao and co-workers reported DFT results of the effect of copper dimer (Cu_2_) embedded in C_2_N on the reduction of CO_2_ to hydrocarbons^35^. Ma and co-workers used DFT to test the embedment of five TM atoms (i.e., Sc, Ti, V, Cr and Mn) in C_2_N for CO and O_2_ oxidation reactions. They concluded that the best catalyst among these to be Cr and Mn^36^. In a related work, Liu and co-workers presented a combination of electrochemical measurements and DFT calculations to study and compare the catalytic activity of MnN_4_ to FeN_4_ embedded in graphene in inducing the oxygen reduction reaction (ORR). They clearly demonstrated the superiority of MnN_4_ catalyst^37^. In a different study based on DFT, Impeng and co-workers investigated the correlation between magnetic moment and strength of chemisorption of 13 gases on MnN_4_-embedded graphene^38^. Only five gases (i.e., NO, NO_2_, O_2_, CO, and SO_2_) exhibited strong chemisorption with large binding energies (-2.30, -1.42, -1.32, -1.11, and -0.51 eV, respectively). Furthermore, only three gases (i.e., NO, CO and NO_2_) were able to reduce the magnetization from 3.01 μ_B_ to 0.13, 1.04, and 2.01 μ_B_, respectively. The estimated recovery times for these three gases were large except CO gas to have 1.7 s. So, the authors proposed MnN_4_-embedded graphene as a platform for a promising magnetic sensor for CO detection^38^. Regarding the magnetism of TM-atom embedded in C_2_N, Du and co-workers presented DFT study of magnetization due the embedment of 3d-TM atoms into the pore of C_2_N. They reported the existence of ferromagnetic ground state for TM atoms: Sc, Ti, V, Cr, Mn, Fe, Co and Ni, and paramagnetic state for Cu and Zn^39^. Wang and co-workers presented DFT study of magnetization in C_2_N nanoribbons (C_2_N-NRs) under the effect of diversifying the passivation of the dangling bonds of the edge atoms^40^. They reported that armchair-edged C_2_N-NR is a non-magnetic semiconductor with direct gap. Whereas zigzag-edged C_2_N-NR is magnetic either semiconductor with indirect band-gap or metallic. Besides the co-saturation of edges with (H and O) or (F and O) can induce a large magnetization into the system to make the system an interesting material for spintronic devices^40^.

The scope of the present work is to study the effect of a magnetic SAC-Mn embedded in C_2_N nanoribbon on the adsorption of seven gases of energy and environmental interest (e.g., CO, CO_2_, H_2_, H_2_O, H_2_S, N_2_ and O_2_) using the state-of-the-art DFT based on VASP. The aim is the attainment of selectivity and analyzing the reasons behind it or its origins, with hypothesis to include among the arguments the inspection of variation of magnetization and charge transfer (i.e., type of molecule whether oxidizing or reducing). The paper is organized as follows: Last section gives details about the model and the computational method. Section 2 gives elaboration of discussing the results. The concluding section summarizes our main findings.

## Results and discussion

### Atomic relaxations

As first stage, the adsorptions of gas molecules on pristine C_2_N nanoribbons were attempted. Atomic relaxations of gas molecules (e.g., CO, CO_2_, H_2_, H_2_O, H_2_S, N_2_ and O_2_) were included in our study. All molecules are found to exhibit physisorption processes on pristine C_2_N-NR and prefer to stabilize above the main pore of the C_2_N-NR at a height of about 2.12, 2.39, 1.61, 1.25, 1.42, 2.30, and 2.0 Å, respectively. In case of angular molecules (e.g., H_2_O and H_2_S) the reported distances are even lower (1.25 and 1.42 Å, respectively) as these molecules are found to stabilize by having their arms directed toward the surface; as likely due to H atoms being electropositive and getting attracted to the electronegative nitrogen atoms of the pore via van der Waals interactions. In the physisorption states of these latter 2 molecules, their angles are found decreased to 101° and 90°; while in the cases of free molecules the angles used to be about 104° and 92°, respectively. Such reductions in angular parameters might be attributed to the transfer of charge from surface to molecules; especially as being oxidizing molecules. However, for the other linear molecules (CO, CO_2_, H_2_, N_2_, O_2_), they seem to stabilize at distances more than 2.0 Å above the surface and in horizontal position parallel to the surface with molecular atoms being close to N-atoms along the direction of the diagonal of the pore.

**Figure. 1:**
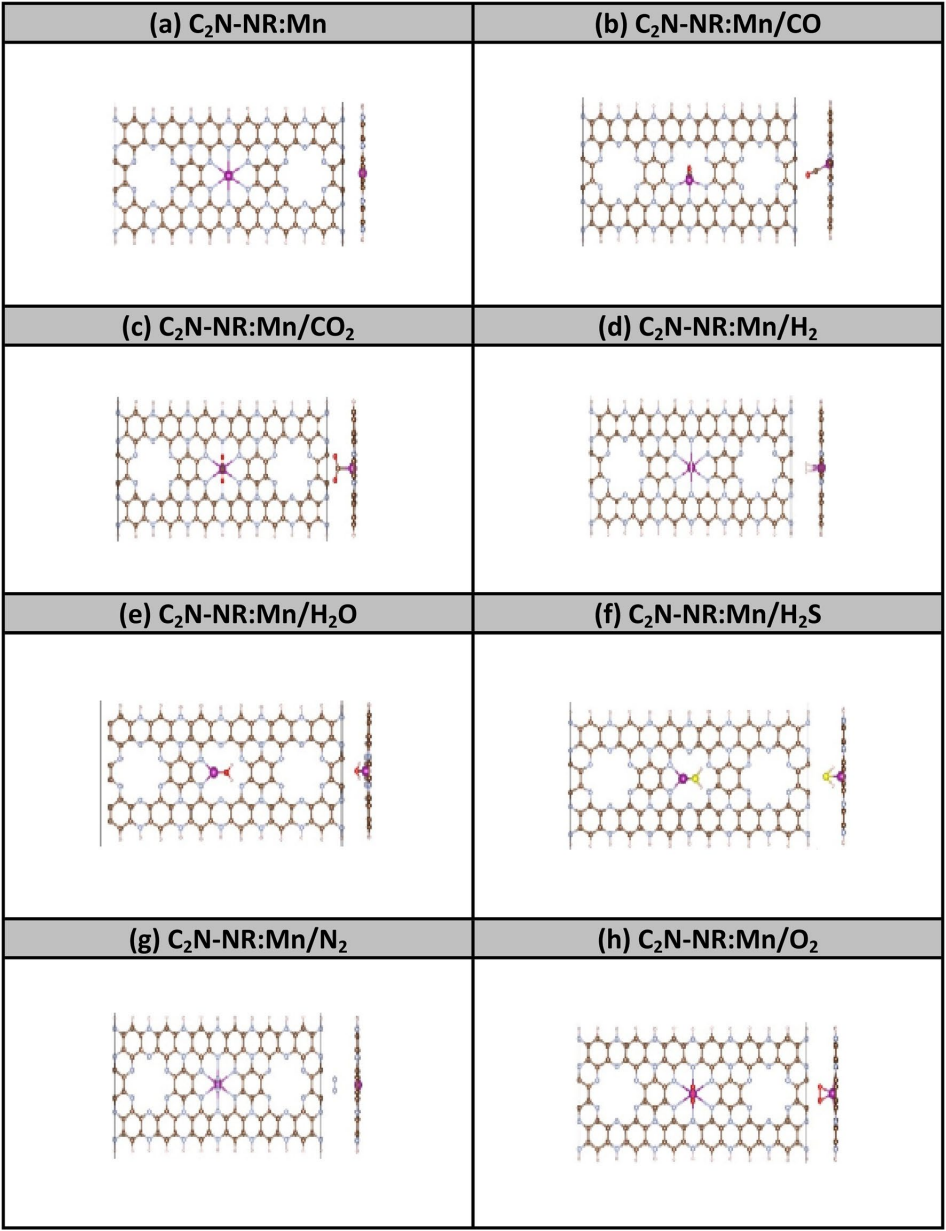
Relaxed geometries of C_2_N-NR:Mn with various gas molecules. Colors: C (brown), N (blue), Mn (purple), O (red), H (yellow), S (orange), and N (grey).

In second stage, the adsorptions of the same gas molecules were attempted on Mn-embedded C_2_N-NRs (i.e., C_2_N-NR:Mn). The results of atomic relaxations are shown in Fig. 1. Chemisorption processes seem to be the destiny to occur for all molecules except N_2_, whose triplet covalent bond seems strong and not dissociable to persist in yielding physisorption process. The N_2_ molecule stabilizes at a distance 3.10 Å above Mn-atom. Table 1 shows the adsorption energy in case of N_2_ to be as low as E_ads_ = -0.123 eV and the molecule to be oxidizing the Mn catalyst with mimic charge transfer of about -0.0178 e. The effect on magnetization is, in turn, very mimic of about 0.027 μ_B_ reduction to corroborate the weak interaction between N_2_ molecule and Mn-catalyst.

**Table. 1:** Adsorption energetics (i.e., E_ads_ = adsorption energy) and magnetization of C_2_N-NR and C_2_N-NR:Mn before and after adsorption of gas molecules. Charge transfer (Δq = q − q_o_), magnetization (M), and change of magnetization (ΔM = M − Mo). All the samples are metallic.

System	E_ad_ (eV)	Δq (e)	M (μ_B_)	ΔM (μ_B_)
C_2_N-NR C_2_N-NR:Mn	N/A N/A	N/A N/A	0 4.868	0
C_2_N-NR/CO C_2_N-NR:Mn/CO	− 0.244 − 1.094	− 0.016 − 0.173	0 4.565	0 − 0.303
C_2_N-NR/CO_2_ C_2_N-NR:Mn/CO_2_	− 0.296 − 0.985	− 0.014 − 0.570	0 3.955	0 − 0.913
C_2_N-NR/H_2_ C_2_N-NR:Mn/H_2_	− 0.093 − 0.194	+ 0.874 − 0.002	0 4.833	0 − 0.035
C_2_N-NR/H_2_O C_2_N-NR:Mn/H_2_O	− 0.059 − 0.188	− 0.015 − 0.021	0 4.949	0 + 0.081
C_2_N-NR/H_2_S C_2_N-NR:Mn/H_2_S	− 0.083 − 0.129	− 0.006 − 0.016	0 4.967	0 + 0.099
C_2_N-NR/N_2_ C_2_N-NR:Mn/N_2_	− 0.213 − 0.123	− 0.014 − 0.018	0 4.841	0 − 0.027
C_2_N-NR/O_2_ C_2_N-NR:Mn/O_2_	− 0.118 − 1.564	− 0.020 − 0.779	2.047 3.587	0 − 1.011

All other studied molecules are found to act as oxidizing the Mn-catalyst. Three molecules (O_2_, CO, and CO_2_) seem to bond strongly to the Mn catalyst causing huge effect on its magnetization more than any other molecule, with reductions of about 1.011 μ_B_, 0.303 μ_B_ and 0.913 μ_B_, respectively. Also, the charge exchanges are drastically large of about -0.779 e, -0.173 e and -0.570 e, respectively. These two indications are signals of potential occurrence of selective gas-sensing of C_2_N-NR:Mn towards these three molecules. It should be emphasized that the chemisorption of O_2_ occurs with partial molecular dissociation as the molecule breaks its π-bond to pave the way for the two oxygen atoms to form new bonds with Mn-catalyst. In case of CO-molecule chemisorption, the bond is formed between Mn-C as carbon atom has ability for four valency. Again, in chemisorption of CO_2_-molecule, the two π-bonds between C-O breaks to pave the way for C-atom to form covalent bond with Mn-catalyst. Consequently, the angle of CO_2_ molecule changes from 180° to 144°.

Relatively weaker chemisorption processes of different style of bonding occur between H_2_-based molecules and the Mn-catalyst embedded in the pore of C_2_N-NR. Panels 1 (e–f) show the relaxed structures of H_2_O and H_2_S on C_2_N:Mn-NRs. Mn-catalyst forms covalent bond with the anionic atom in the molecule with bond-length d(Mn–O) = 1.99 Å and d(Mn-S) = 2.44 Å. Specifically, the O-atom has stronger bond with Mn than does S-atom because it has more electronegativity (i.e., χ^O^ = 3.44 Pauling > χ^S^ = 2.58 Pauling). The hydrogen arms of molecules are directed closer to the surface as could be attributed to the molecular dipole interaction with the surface dipole moments. The effects of these two bonding structures on the charge transfer and magnetization are similar but smaller compared to the previous group of molecules (i.e., O_2_, CO, CO_2_). The chemisorption of H_2_ molecule, shown in Panel 1(d), has similar mimic effects on Δq and ΔM and, thus, should belong to this group. The bond length d(H–H) = 0.76 Å is a bit larger than the free molecule. The molecule looks like it did not split because the calculation is carried out at 0 K under the assumption of frozen-lattice and the validity of the Born–Oppenheimer approximations.

### Spin-polarized band-structures

**Figure. 2:**
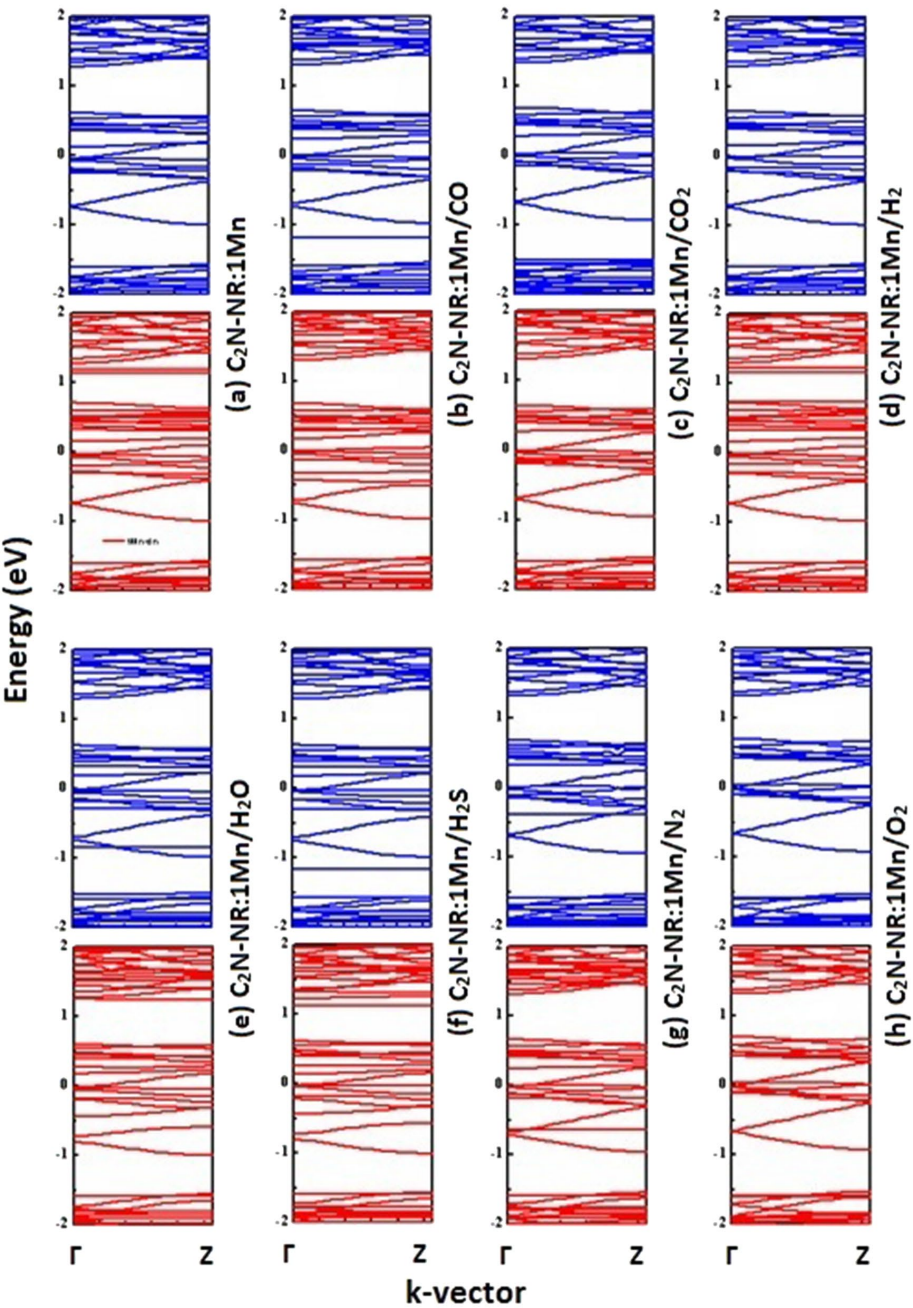
Electronic band structures of C_2_N-NR:1Mn after adsorption of various gas molecules. Spin-up (down) bands are shown by blue (red) color.

Figure 2 displays the spin-polarized band structures of Mn-embedded in C_2_N-NR and its interactions with the 7 studied molecules in same order as been presented in Fig. 1 with Fermi level taken as an energy reference. One can observe the followings trends: (1) Before the arrival of any gas molecule, the band structures of C_2_N:Mn show great discrepancy between spin-up and spin down as shown in Fig. 2(a). Single-atom catalyst Mn introduces a large magnetization in the lattice of about M = 4.868 μ_B_. (2) Case of N_2_ physisorption: Fig. 2(g) shows the mimic effects of weak interaction of N_2_ with C_2_N:Mn on the band structure. Basically, one can notice the introduction of two discrete states (i.e., one localized state at energy E ≈ -0.4 eV in the spin-up bands and another one at energy E = -0.75 eV in the spin-down bands); which should be attributed to the non-bonding states of the molecular orbital of N_2_ molecule. (3) Strongly chemisorbed molecules (CO, CO_2_ and O_2_): These three molecules exhibit strong chemisorption processes with C_2_N:Mn as was evidenced by the large charge transfers shown in Table-1 and was explained in details in sub-Sect. 3a in discussing results of Figure-1. Their corresponding band structures (see Panels 2b, 2c and 2 h, respectively) show the evidence of enormous changes to occur in the spin-down band structures. For more details, one must deal with density-of-states perspectives (e.g., see next sub-sections). Nevertheless, these modifications happening in the spin-down band-structures might be originated from the huge reductions in magnetization (see Table-1) of values ΔM = -0.303 μ_B_, -0.913 μ_B_ and -1.011 μ_B_, respectively. (4) Weakly chemisorbed molecules (H_2_O, H_2_S, H_2_): Their corresponding band-structures (see Panels 2e, 2f. and 2d, respectively) show less affected bands than those of the previous group of molecules. Basically, one can notice some localized states at energies E ≈ -0.85 eV and E ≈ -1.2 eV introduced in the spin-up bands by H_2_O and H_2_S molecules, respectively. Besides these effects, small changes might be introduced by all these molecules (H_2_O, H_2_S, and H_2_) in the spin-down bands. Consequently, the effects of these molecules interacting with the C_2_N:Mn lattice on the magnetization are mimic of order + 0.081 μ_B_, + 0.099 μ_B_ and – 0.035 μ_B_, respectively.

### Spin-polarized density of states

**Figure. 3:**
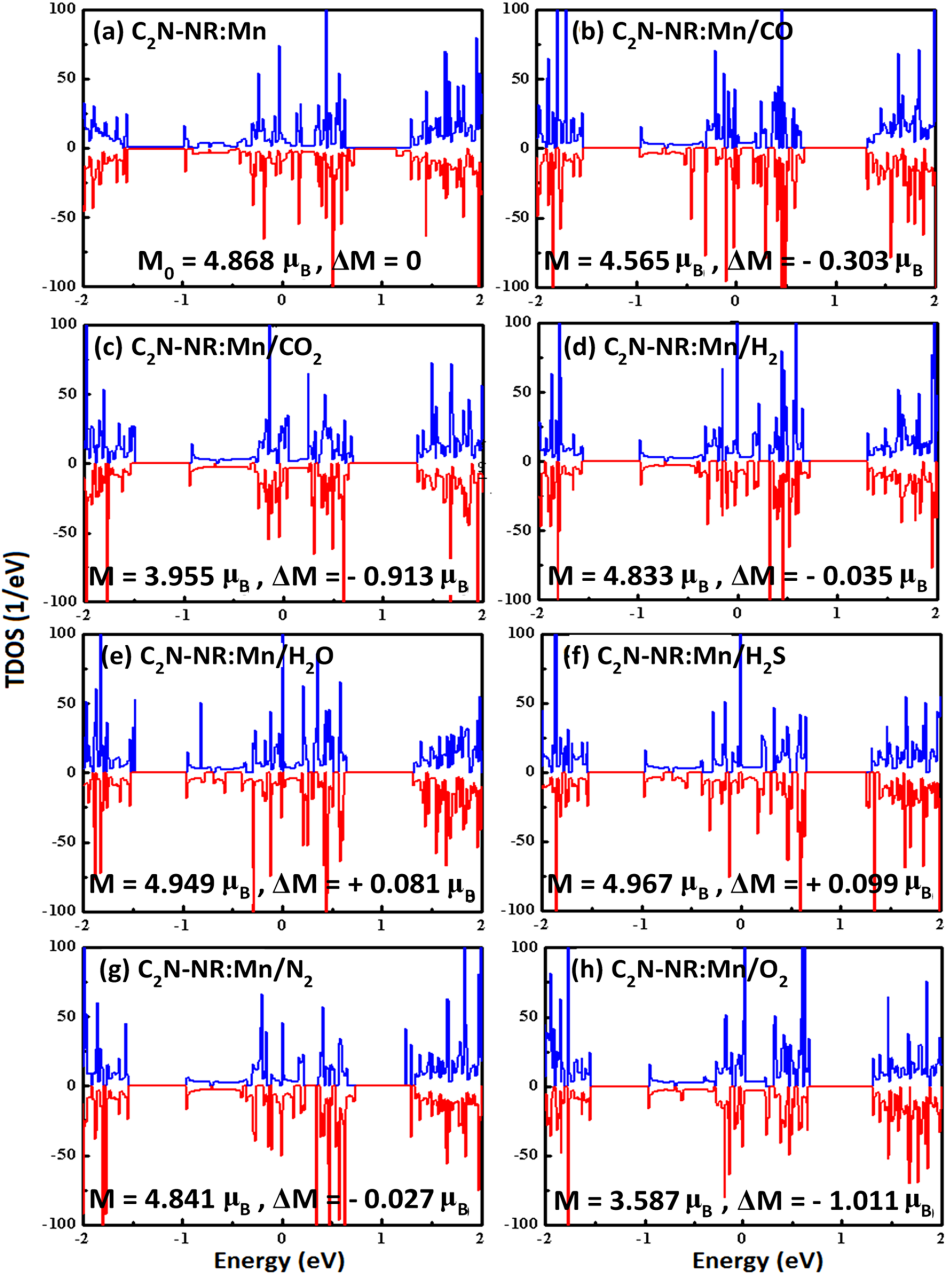
Spin-polarized Total Density of States (TDOS) for C_2_N-NR:1Mn with various gas molecules.

Figure 3 displays the spin-polarized total density of states (TDOS) for the same systems studied in Fig. 2. The spin-up (down) bands are shown in blue (red) color. The Fermi energy is taken as an energy reference (E_F_ = 0). The common trend between all panels is that they all show not only metallic behavior but also the existence of magnetization, as revealed by the existence of the discrepancy between TDOS of spin-up and TDOS of spin down in each single panel. The value of magnetization in units of Bohr magneton (μ_B_) is displayed in each panel. The difference of magnetization is defined as ΔM = M_g_-M_o_, where M_g_ and M_o_ are total magnetization with and without gas molecule, respectively. The starting magnetization of the substrate (i.e., corresponding to C_2_N:Mn without gas) is calculated to be M_o_ = 4.868 μ_B_. The discrepancy between two TDOSs of spin-up and spin-down should be proportional to the magnitude of magnetization. By taking C_2_N:Mn without gas molecule as a reference of magnetization, then ΔM should be a measure of the variation of that discrepancy. Figure 3 does corroborate the results of magnetization in measuring the effect of adsorption on the electronic structure in case of existence of magnetic dopant such as our Mn-catalyst. So, one can categorize three groups: (1) Case of physisorption (N_2_ molecule): This adsorption has the least effect as revealed by the small decrease in magnetization ΔM = -0.027 μ_B_ . (2) Strongly chemisorbed molecules (CO, CO_2_, O_2_): These adsorptions have the largest effects on both electronic structure and magnetization. The variation of magnetization recorded to be ΔM = -0.303 μ_B_, -0.913 μ_B_, and -1.011 μ_B_, respectively. (3) Weakly adsorbed molecules (H_2_O, H_2_S, H_2_): Here the magnetization varies within few hundredths of Bohr magneton (i.e., -0.035 μ_B_, + 0.081 μ_B_, and + 0.099 μ_B_, respectively). In order to further investigate the origin of the discrepancy (i.e., ΔM), one needs to dig into the spin-polarized partial and orbital density of states that we do next.

### Magnetization effects

**Figure. 4:**
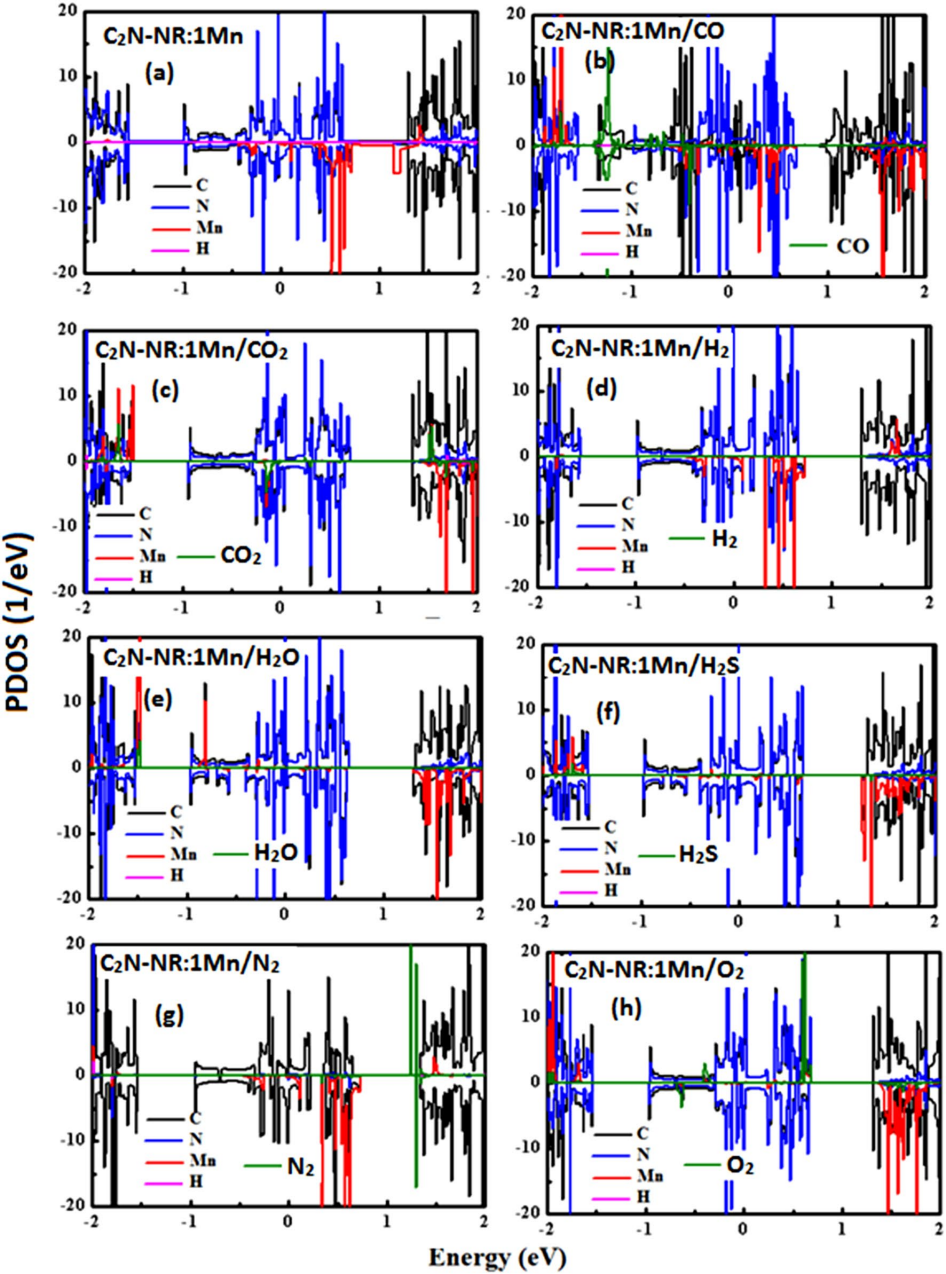
Spin-polarized Partial density of States (PDOS) for C_2_N-NR:Mn with various gas molecules.

To inspect the effects of magnetization of Mn-catalyst when embedded into the pore of C_2_N-NR, one needs to carry on further calculations such as the spin-polarized partial density of states (PDOS) and orbital density of states (ODOS). Figure 4 displays the results of spin-polarized PDOS on same systems presented in previous figure. In each panel, spin-up and spin-down contributions to PDOS are presented on the positive and negative sides, respectively. Moreover, the PDOS are clearly shown to have many peaks which correspond to Van-Hove singularities as characteristics of DOS of 1D systems. It is worth eliciting that in all panels hydrogen contributions should be expected as the hydrogen atoms were used to saturate the dangling bonds at the NR-edges. It is further clearly noticed the existence of asymmetry between spin-up and spin-down contributions whose discrepancy should account for the formation of magnetization. First, one should start describing the case of the substrate or the platform to be used for the gas detection or sensing.

Panel 4(a) shows the case of C_2_N-NR:Mn before the arrival of any gas molecule. Mn-catalyst’s PDOS (in red color) is shown to have clearly huge asymmetry with large contribution from spin-down states. Mn is located at the pore center and that should be considered to be the center of the magnetization. Mn-catalyst would induce some magnetization to neighboring atoms (i.e., so called “spin relaxation”). So, N-atoms’ PDOS (blue color), in turn, shows some asymmetry to reveal its participation to the collective magnetization. C-atoms’ PDOS (in black color) is the lease affected but does show some asymmetry in its overall profile especially above Fermi level. Now, one should discuss the effects of interactions of lattice (platform) with various gas molecules. In panel 4(b): CO molecule’s PDOS (in green color) show great asymmetry contributions between spin-up and spin-down states. Near Fermi level, one can notice more contribution from spin-up states in response to the bonding of C-atom to Mn-catalyst due to the occurrence of chemisorption. So, one should expect some reduction of magnetization (i.e., ΔM = -0.303 μ_B_). In panels 4(c,h): CO_2_ and O_2_ molecules’ PDOSs (in green color) show even stronger effect than CO-molecule. Large contribution is due to spin-up states and causing large reductions in magnetization (i.e., ΔM = -0.913 μ_B_ and -1.011 μ_B_, respectively). In panels 4(d,e,f): H_2_, H_2_O and H_2_S molecules’ PDOSs (in green color) are basically overlapped to follow the lattice profile. So, the asymmetry is not pronounced as in previous case and consequently the effect on magnetization is mimic (i.e., |ΔM|= 0.03–0.09 μ_B_). The last panel to discuss is panel 4(g): N_2_ molecule exhibits physisorption process so that its PDOS shows discrete molecular levels (i.e., see 2 spin-up states at energies E =  + 1.2 eV and + 1.3 eV, and one spin-down state at energy E =  + 1.3 eV). Yet somehow, small magnetic moment got induced into the N_2_ molecule by its being close above Mn-catalyst at a distance of about 3.32 Å (i.e., with ΔM = -0.027 μ_B_).

**Figure. 5:**
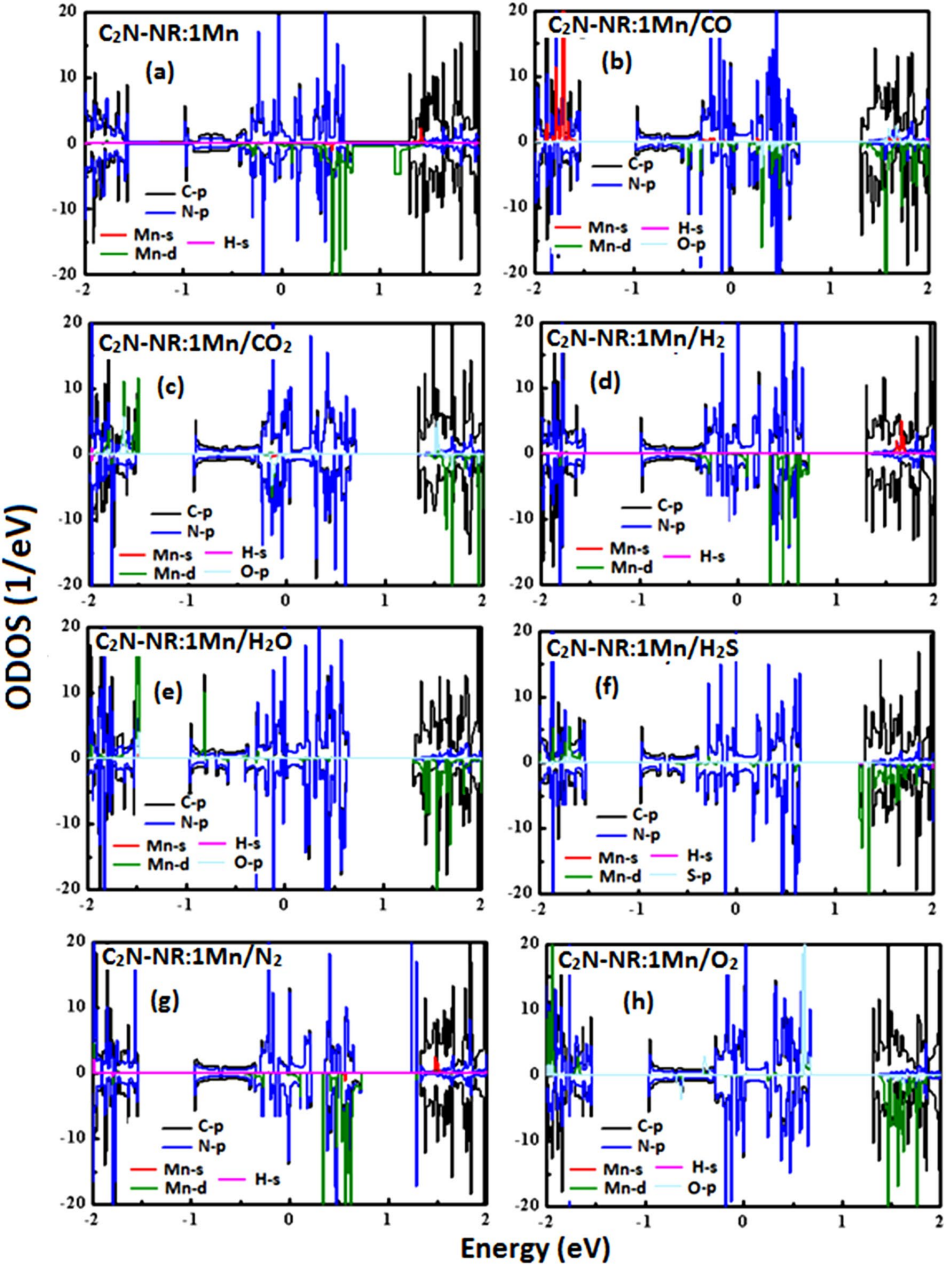
Spin-polarized Orbital density of states (ODOS) for C_2_N-NR:Mn with various gas molecules.

Figure 5 shows the results of ODOS on the same systems studied in previous figure. This figure should investigate the original atomic orbital contributing to magnetization or causing effect on it. Panel 5(a) shows the case of our substrate/platform (i.e., C_2_N-NR:Mn). The valence electrons in Mn-catalyst reside at s and d-states. Panel 5(a) shows that d-states have huge contribution to magnetization by having huge discrepancy between spin-up and spin-down ODOSs. N and C atoms are forming the lattice with planar hybridization sp^2^ while their ${P}_{z}$-orbitals populate the states at Fermi level and are accessible to be perturbed under the effect of magnetization; especially if this latter is attributed to z-based d-orbitals (i.e., ${d}_{yz},{d}_{zx},{d}_{{z}^{2}}$). So, we decided to present only P-orbitals of C, N, S and O atoms to assess their contributions to magnetization (with PDOS in light-blue color). Panels 5(b,c,h) show the cases of strong chemisorption processes with CO, CO_2_ and O_2_ molecules. It seems that the formation of an axial σ-bond between the molecule and Mn-catalyst would impose on the molecule to get its electronic contributions from spin-up states because Mn-catalyst has mostly spin-down states near Fermi level. This is to fulfill the requirements of Pauli-exclusion principle. In doing such act and having strong covalent bond would reduce the discrepancy in electronic population between spin-up and spin-down. Consequently, such Pauli exclusion principle effect would cause large reductions in magnetization. Panels 5(d,e,f) correspond to the weak chemisorption processes of H_2_, H_2_O and H_2_S molecules, respectively. The chemical bonds here are weaker and Pauli-exclusion principle is less effective. So, the discrepancy between spin-down and spin-up PDOSs are less pronounced than those of the previous group of molecules. So, these latter chemisorption processes cause less effect on magnetization. Last case is in Panel 5(g) corresponding to the physisorption of N_2_ molecule. The molecular states are discrete and having less discrepancy between spin-up and spin-down states. So, the effect on magnetization is the least one.

### Charge transfer

**Figure. 6:**
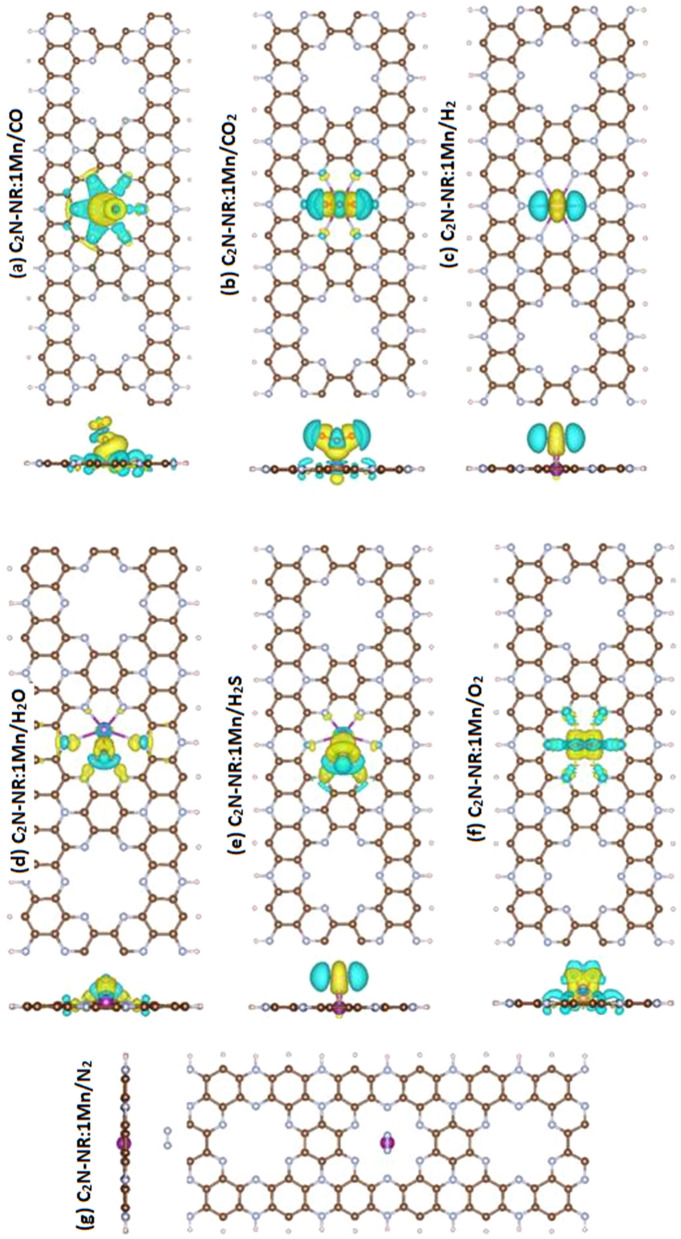
Charge-difference density plots for C_2_N-NR:1Mn with various gas molecules. Yellow (cyan) color indicates charge depletion (accumulation).

Figure 6 displays the difference in charge density plot between the cases after the adsorption and before the adsorption for 7 molecules adsorbed on C_2_N-NR:Mn substrate in the following order: (a) CO, (b) CO_2_, (c) H_2_, (d) H_2_O, (e) H_2_S, (f) O_2_, and (g) N_2_ molecules. To study the charge-transfer process between molecule and substrate/platform, it is customary to explore one of three methods: (i) Compare the charge contour plots before and after adsorption; or (ii) Compare the plots of the highest occupied molecular orbital (HOMO) and the lowest unoccupied molecular orbital (LUMO) before and after adsorption; or (iii) Do everything in one plot illustrating the charge difference/transfer such as the one shown in Fig. 6, where yellow (cyan) color indicates charge depletion (accumulation).

In all cases, the 7 molecules are oxidizing the Mn-catalyst. In the 6 cases of chemisorption, the molecule is attached to Mn-catalyst via covalent bond with partial ionic character. Panels 6 (a,b) show that the cases of chemisorption of CO and CO_2_ molecules to be associated with the depletion of charge from vicinity of Mn and its shifting toward C and O in the molecule as well as the 6 N atoms neighboring Mn. The discrepancy in electronegativity characters between the atoms play a major role in the charge distribution along the bond. For instance, χ^O^ = 3.44 > χ^N^ = 3.04 > χ^C^ = 2.55 > χ^Mn^ = 1.55 (in units of Pauling) can be used to justify the accumulation of charge at the sites of anion atoms of higher electronegativity. Panel 6(c) shows the case of chemisorption of H_2_ to be accompanied with the transfer of charge toward H atoms because χ^H^ = 2.20 > χ^Mn^ = 1.55 (in Pauling units). Furthermore, the charge distribution clearly shows the H-atoms to be well separated confirming the occurrence of chemisorption associated with a weak molecular dissociation. Panels 6(d,e) show that the H_2_O and H_2_S molecule maintain their molecular structures while the anion atom is making somehow weak covalent bonding with Mn-catalyst (i.e., so less ΔM occurs). Panel 6(f) demonstrates a kind of strong chemisorption with O_2_ molecule with strong charge transfer to it. Last panel 6(g) shows the case of N_2_ physisorption. It is clearly shown almost inexistence of charge transfer between molecule and substrate.

**Figure. 7:**
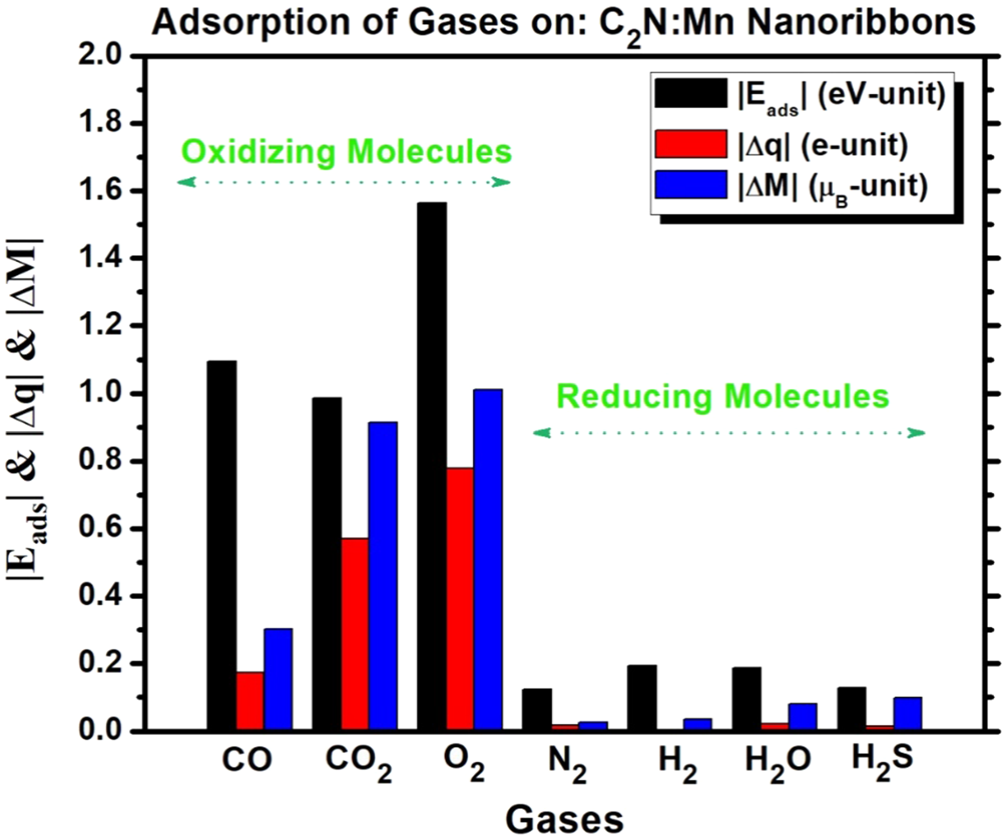
Comparison of absolute values of adsorption energy (|E_ads_|), charge transfer (|Δq|) and variation of magnetization (|ΔM|) from before to after the adsorption of gas molecules.

Figure 7 summarizes our findings and displays the results of Table-1 concerning the absolute values of adsorption energy, charge transfer, and change in magnetization. Clearly, one can distinguish two groups of molecules in the perspective of their interactions with the SAC-Mn embedded in C_2_N-NR: (i) A first group with strong and well-pronounced oxidizing character (e.g., CO, CO_2_ and O_2_) with strong chemisorption: These molecules have strong adsorption energy. They make strong covalent bonds with SAC-Mn and thus introduce spin-up states to TDOS in the Fermi-energy region and consequently reduce the discrepancy between spin-up and spin-down TDOS. Thus, they are able to reduce enormously the net magnetization. (ii) A second group with vanishing oxidization or rather known to have reducing character (e.g., N_2_, H_2_, H_2_O, and H_2_S) with either physisorption or weak chemisorption: These molecules have weak interactions with SAC-Mn. Thus, they contribute mostly with spin-down states to TDOS in the Fermi-energy region of same kind as the magnetic Mn-atom. So, the discrepancy between spin-up and spin-down TDOS remains about unchanged and the effect on changing the magnetization would be vanishingly small (i.e., the magnetization is maintained robust or conserved). So, one can conclude that C_2_N:Mn would be a good candidate for platform in the fabrication of magnetic sensor with promising selectivity to detect the oxidizing gas molecules (CO, CO_2_ and O_2_).

## Conclusions

Aiming for Gas-sensing selectivity, we presented a theoretical study of functionalized C_2_N nanoribbons based on the DFT-package of VASP. Magnetic atom such as Mn is used as SAC embedded in C_2_N pore to test the effect on the adsorption properties of various gases of interest in energy and environmental sciences (e.g., CO, CO_2_, H_2_, H_2_O, H_2_S, N_2_ and O_2_). The results of atomic relaxations show that pristine C_2_N-NR always alters physisorption processes with these gases while C_2_N-NR:Mn has the full ability to alter chemisorption processes with all gas molecules except N_2_. The results of molecular chemisorption can be categorized into two groups:

- Strong chemisorption with oxidizing CO, CO_2_, and O_2_ gas molecules is evidenced by large binding energy and charge transfer. Mn-catalyst seems very active in interacting with the oxidizing gas molecules. Furthermore, SAC-Mn induces large magnetization into the system by contributing enormously with spin-down states to the electronic band-structure at Fermi-energy region, while its interaction with these mentioned molecules would introduce spin-up states at Fermi level to compromise and rather reduce the magnetization. Actually, such phenomenon occurs as a consequence of the Pauli-exclusion principle;
- Weak chemisorption with reducing H_2_, H_2_O, and H_2_S gas molecules is observed with low binding energy and charge transfer. The interaction does not affect the asymmetry in spin-up and spin-down statistics and the discrepancy remains about the same between the states of spin-up and spin-down. Consequently, these adsorptions have mimic effects on magnetization (i.e., M persists to remain constant).

The results suggest that C_2_N-NR:Mn is a promising platform for gas sensing of oxidizing CO, CO_2_ and O_2_ gas molecules with high sensitivity and selectivity. The efficiency of the sensor could be further enhanced if it is combined with magnetic sensor to detect the change in magnetization and the system would be of great importance in environmental applications.

### Computational methodology

Density functional theory (DFT) has been well established to be the most reliable to predict the ground state properties of materials, including the adsorption properties. Perhaps, the worldwide most popular and reliable package is the Vienna Ab-initio Simulation Package (VASP)^41^, which masters to incorporate all basic interactions, such as spin-polarization, magnetic (Hubbard U) and dipole–dipole long-range (i.e., van der Waals) interactions. Furthermore, the package is competent to deal with challenges in our system which involves magnetic dopants (e.g., manganese “Mn”) and its binding as embedded in pore of C_2_N as well as its interaction with gas molecules in order to reliably predict the occurrence of either physisorption or chemisorption. Our calculations include atomic relaxation to study the adsorption of various gas molecules, known to be either oxidizing (e.g., CO, CO_2_, O_2_) or reducing (e.g., H_2_, H_2_O, H_2_O, N_2_), spin-polarized band structures and partial as well as orbital densities of states, difference of charge density plots, Bader-charge analysis, and magnetic moments. Targeting to use such system of Mn-embedded in C_2_N as a platform of electrical gas sensor, we decided to study the system in the nanoribbon form^40^.

As a model, we have designed a nanoribbon shape with zigzag edges and made the dangling bonds be saturated at the two edges by bonding them with hydrogen atoms. Actually, such a structure was used before us and exists in literature^40,41^. We made sure that it is thermodynamically stable and it yields metallic behavior (i.e., Eg = 0), to make it suitable for gas-sensing applications. The designed C_2_N-NR structures are displayed in Figure-1. We emphasize that the primitive cell of pristine C_2_N-NR is composed of 50 atoms (i.e., 32 C + 10 N + 8 H atoms). The supercell is composed of 3 primitive cells so the total number of atoms is 150 atoms (i.e., 96 C + 30 N + 24 H atoms). Thus, in summary, our supercell would contain 1D nanoribbon having a periodicity along x-axis with length L = 29.51 Å and a width of approximately about W ≈ 12 Å, and 3 pores each of size/diameter of about 5.68 Å. Given the size of the C_2_N-NR, our computational supercell was set to the dimensions (A,B,C) = (29.51, 22, 12) Å units. The B and C dimensions are large enough to ensure the isolation of C_2_N-NR with its mirror symmetries along y and z directions, respectively. Throughout our present investigation, we used as adsorbent either (1) Pristine C_2_N-NR, or (2) SAC-Mn embedded in the central pore of C_2_N-NR; and as adsorbate one gas molecule at a time among the 7 gases mentioned above.

As a method, the calculation employs plane-wave basis set (with cutoff energy of 500 eV). Within the framework of the projected-augmented plane-waves (PAW) method, the electronic exchange–correlation was treated using the generalized gradient approximation (GGA + U)^43^, where + U stands for the Hubbard parameter which is usually added in case of highly-correlated spin systems such as transition metal, and especially those which are ferromagnetic such as in our case “Mn”. The Hubbard parameter U = 3.5 eV was taken for Mn 3d-states was due to reference 43. Furthermore, we included in our calculations DFT-D3 technique^44,45^ to take care of the van der Waals interactions, which are important in the study of adsorption. In the geometry optimization, all the atoms in the supercell were allowed to relax until the Hellmann–Feynman force on each atom became smaller than 0.01 eV Å^−1^; whereas the tolerance for the total energy convergence was set to 10^–4^ eV. The sampling of the Brillouin zone was performed using 25 × 1 × 1 Monkhorst–Pack technique^46^ for total energy calculations. However, the density of states’ calculations were performed with a relatively denser grid of 50 × 1 × 1 k-mesh. The adsorption energy of any gas molecule on C_2_N-NR substrate was evaluated using the formula:

1 $$E_{ads} = E_{{C_{2} N - NR + gas}} - E_{{C_{2} N - NR}} - E_{gas} ,$$

where $E_{{C_{2} N - NR + gas}}$, $E_{{C_{2} N - NR}}$, and $E_{gas}$ are the total energies of the system of pristine (or Mn-embedded) C_2_N-NR with and without gas molecule and the gas molecule, respectively. Furthermore, we emphasize that the charge transfer was estimated using the Bader-charge analysis^47^.

In order to address the magnetic coupling, and to find the most stable magnetic state, a dimer of Mn is embedded in the pore of C_2_N-NR (i.e., C_2_N-NR:2Mn) and the system is relaxed under ferromagnetic (FM) and anti-ferromagnetic (AFM) spin configurations. The obtained results of total energy and magnetization are summarized in Table 2.

**Table. 2:** Total energy and magnetization and separate magnetic moments of Mn dimer embedded in C2N. Two spin configurations (FM and AFM) were tested.

Spin Configuration	E_TOT_ (eV)	M_TOT_ (μ_B_)	M/Mn-atom: Mn_1_, Mn_2_ (μ_B_)	Mn–Mn Distance	Favored Coupling
FM	− 1210.504	7.010	3.099, 3.101	2.207	No
AFM	− 1211.099	0.000	3.458, − 3.458	2.178	Yes

The results were in favor of anti-ferromagnetic state as having lower total energy by ΔE = E_tot_(FM)-E_tot_(AFM) =  + 0.595 eV value. Nevertheless, in our present investigation, the focus will not be upon the dimer-atom-catalyst (DAC) case but the full focus will be upon the single-atom-catalyst (SAC) case. So, we study single Mn atom embedded in C_2_N-NR and assess its interaction with various gas molecules. Definitely, we expect non-zero magnetic moment and rather large effect of magnetization on the electronic structures as will be demonstrated by the spin-relaxation effects.
